# Building Support for Adolescent Sexuality and Reproductive Health Education and Responding to Resistance in Conservative Contexts: Cases From Pakistan

**DOI:** 10.9745/GHSP-D-17-00285

**Published:** 2018-03-21

**Authors:** Venkatraman Chandra-Mouli, Marina Plesons, Sheena Hadi, Qadeer Baig, Iliana Lang

**Affiliations:** a Department of Reproductive Health and Research/Human Reproduction Programme, World Health Organization, Geneva, Switzerland.; b School of Public Health, University of Washington, Seattle, WA, USA. Now with Department of Reproductive Health and Research/Human Reproduction Programme, World Health Organization, Geneva, Switzerland.; c Aahung, Karachi, Pakistan.; d Rutgers Pakistan, Islamabad, Pakistan.; e Georgetown University, Washington, DC, USA.

## Abstract

**Background:**

Despite international recommendations and supportive evidence, there are few examples of scaled-up and sustained programs to provide adolescents with sexuality education. Moreover, despite acknowledgment that building community support and responding to resistance are key challenges, there is a lack of detailed discussion on specific programmatic strategies to address these issues.

**Objectives:**

This article reviews the work of 2 organizations—Aahung and Rutgers Pakistan—that are successfully implementing large-scale sexuality education programs in Pakistan, collectively reaching more than 500,000 students. This review aims to answer the following questions: (1) How did Aahung and Rutgers Pakistan work to understand Pakistani society and culture and shape their programs to build community support? (2) How did Aahung and Rutgers Pakistan overcome resistance to their efforts?

**Methods:**

We reviewed program documents and publications, synthesized key themes, identified questions of interest, and engaged key informants from Aahung and Rutgers Pakistan's leadership.

**Results:**

The success of Aahung and Rutgers Pakistan was grounded in their readiness to understand the nuanced context within the communities, collaborate with groups of stakeholders—including parents, school officials, religious leaders, media personnel, and adolescents themselves—to ensure support, and stand up to forces of resistance to pursue their goals. Specific strategies included working with communities to select content, tactfully selecting and framing issues with careful consideration for sensitivities, engaging adolescents' influencers, strengthening media presence, showcasing school programs to increase understanding and transparency, and choosing opportune times to introduce messages.

**Conclusion:**

The successful strategies used by Aahung and Rutgers Pakistan to promote adolescent sexual and reproductive health through sexuality education can inform programs worldwide. Additionally, the programmatic weaknesses identified can guide future planning and action by Aahung and Rutgers Pakistan. We call on other programs to continue sharing challenges, specifically related to resistance, with sexuality education programs in order to develop a toolbox of additional strategies for community uptake.

## Abstract (precis)

While there is no one-size-fits-all approach to building community support for such programs, key strategies in Pakistan included: (1) sensitizing and engaging key stakeholders, including religious groups, schools, health and education government officials, parents, and adolescents themselves; (2) tactfully designing and framing the curricula with careful consideration of context and sensitive topics; (3) institutionalizing the programs within the school system; (4) showcasing school programs to increase transparency; and (5) engaging the media to build positive public perceptions.

## INTRODUCTION

Pakistan has long been a challenging setting for the promotion of adolescent sexual and reproductive health (SRH).^1^^,^^2^ As in many other countries worldwide, there is little acknowledgment that adolescents have sex, whether consensual or coerced, before marriage and many believe that exposure to sexuality education will incite unwanted behavior. Furthermore, despite the fact that many adolescent girls marry early, there is also little acknowledgment that married adolescents need to be proactively prepared to meet their SRH needs and promote their well-being.^3^

Two NGOs in Pakistan—Aahung and Rutgers Pakistan (the Pakistani chapter of Rutgers)—have developed effective strategies to support adolescents' knowledge and understanding of SRH, and in some cases empowerment, in this context (Box 1). Working around the social and religious barriers to sexuality education, Aahung and Rutgers Pakistan have skillfully crafted and implemented large-scale sexuality education programs in Pakistan, collectively reaching more than 500,000 students.

BOX 1 **What Are Aahung and Rutgers Pakistan?**

**Aahung**

Aahung is a Pakistani organization that has been working to support girls' and boys' sexual and reproductive health and rights in Pakistan's Sindh province since 1995. Over the past 8 years, Aahung has institutionalized life skills-based education by operating within the school system and by working directly with teachers to strengthen capacity on participatory and learner-centered methodologies. Its curriculum, which introduces critical health information and management skills in line with the emerging capacity of adolescents, has been implemented in 196 schools, training 1,946 teachers and ultimately reaching more than 200,000 students.^4^

**Rutgers Pakistan**

Rutgers Pakistan (founded in 1997) is the Pakistani chapter of Rutgers, a Dutch organization that operates internationally with extensive expertise on sexual and reproductive health and rights in the Netherlands, Africa, and Asia. Rutgers Pakistan coordinates 3 life skills-based education programs—Access, Services and Knowledge; Unite for Body Rights; and dance4life—to empower young people to achieve and safeguard their sexual and reproductive health and rights. Their school-based adolescent education program has reached a total of 1,188 schools and 312,807 students.^5^

While there are numerous descriptions of projects and programs on sexuality education, there is a lack of research and discussion on successful strategies to create support for and overcome resistance to its implementation in schools and communities.^6^^–^^8^ The landmark1994 International Conference on Population and Development called on countries to educate young people about SRH using age-appropriate and context-specific content and strategies.^9^ However, since this call, organized resistance and misconceptions about sexuality education have challenged efforts. A 2014 report from the United Nations Educational, Scientific and Cultural Organization (UNESCO) noted that there are few examples of scaled-up and sustained programs on these issues.^10^ The growing body of evidence on the scale-up of sexuality education programs has noted resistance as a challenge, but it has not provided a detailed discussion of the nature of resistance or strategies to overcome them.

There is a lack of research on strategies to create support for and overcome resistance to sexuality education.

To fill this gap, we examined the strategies used by Aahung and Rutgers Pakistan to design and implement sexuality education programs in Pakistan. This analysis will not examine the programs' coverage, quality and fidelity, the reactions of young people, or the effects of the programs on knowledge, understanding, behaviors, or health, as these topics have been documented elsewhere.^4^^,^^5^^,^^11^ Instead, we guided our analysis of Aahung and Rutgers Pakistan's programs on sexuality education with the following 2 questions:

1. How did Aahung and Rutgers Pakistan work to understand Pakistani society and culture and shape their programs to build community support?
2. How did Aahung and Rutgers Pakistan overcome resistance to their efforts?

## METHODS

To address these 2 questions, we drew from Aahung and Rutgers Pakistan's program documents and publications to synthesize information and extract strategies for building community support, overcoming resistance, and advancing sexuality education. Building on this review, we identified questions of interest and engaged key informants from the leadership of Aahung and Rutgers Pakistan. We conducted interviews with Sheena Hadi, the executive director of Aahung, and Qadeer Baig, the country representative for Rutgers Pakistan. Their expert testimonies provided critical insights and allowed for a focused discussion on Aahung and Rutgers Pakistan's experiences of building community support and overcoming resistance.

## FINDINGS

### Pakistan in Context

The World Economic Forum's *Global Gender Gap Report 2016* ranked Pakistan 143 (out of 144).^12^ Compared with their male counterparts, women in Pakistan typically have little to no decision-making power, fewer educational opportunities, and less control over assets and resources.^4^ Perhaps unsurprisingly, the country has long been a challenging setting for programs relating to sexuality education, reproductive health, youth engagement, and women's empowerment. Young people, who comprise 21% of Pakistan's total population, face numerous challenges related to their SRH, including high rates of early marriage and pregnancy, sexual violence, and risk behaviors such as substance use.^13^ Meanwhile, schools rarely include SRH content in their curricula, lack of knowledge and misconceptions about SRH are common, and adolescent-friendly SRH services are largely absent in the public sector.^4^^,^^5^ Religious resistance is commonly identified as a major barrier to large-scale, and particularly school-based, sexuality education programs. Additionally, the 2010 decentralization of policy-making power in key areas such as health, education, and social welfare to the provincial level has posed a significant challenge to the large-scale implementation of sexuality education programs.^5^

Young people in Pakistan face numerous challenges related to their sexual and reproductive health.

Instead of viewing such opposition as an insurmountable barrier to sexuality education initiatives, Aahung and Rutgers Pakistan have created strategies to work within this context to improve the SRH and developmental well-being of young people in Pakistan. Operating independently, but as part of an informal network of NGOs working on adolescent SRH in Pakistan, Aahung and Rutgers Pakistan are running sexuality education programs in all 4 provinces of Pakistan, reaching a total of more than 500,000 students, with geographic coverage concentrated in the provinces of Punjab and Sindh.

### How Aahung and Rutgers Pakistan Shaped Their Programs to Build Community Support

**FIGURE 1.**
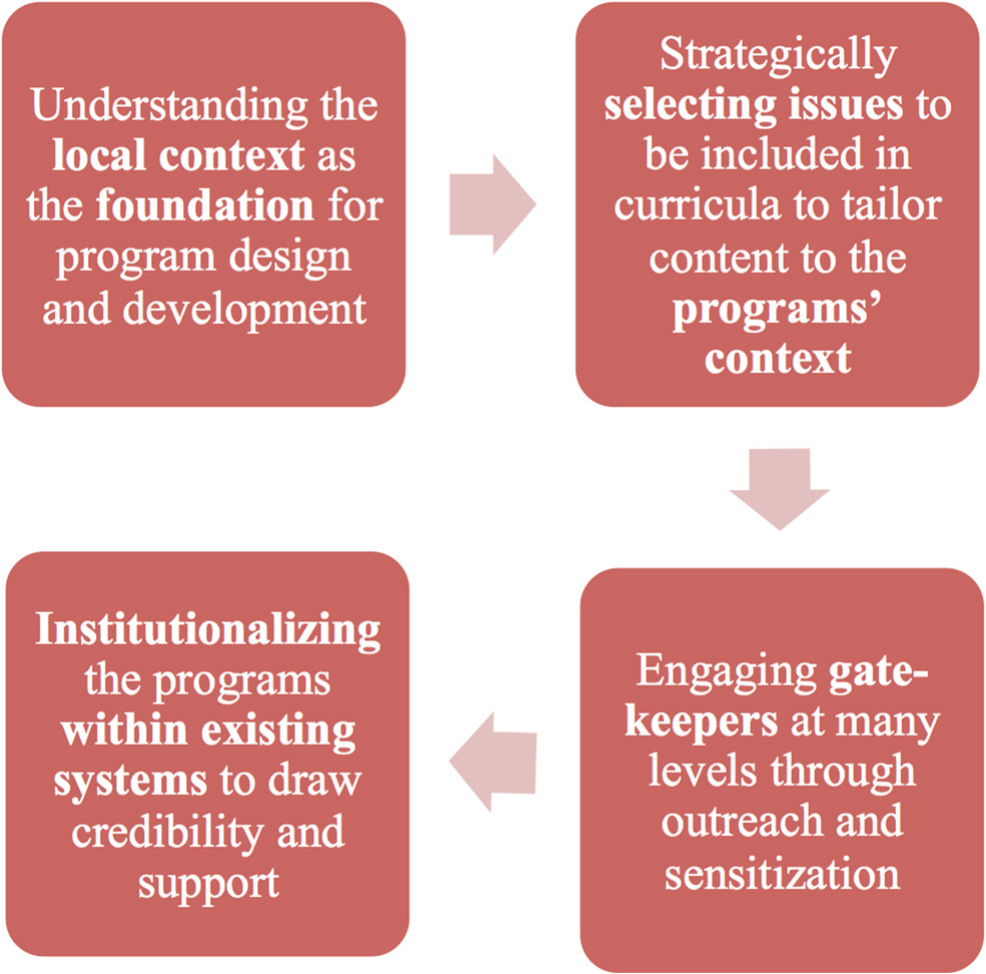
Strategies Used by Aahung and Rutgers Pakistan to Build Community Support for Their Sexuality Education Programs

Acknowledging deep-rooted societal and cultural barriers, both Aahung and Rutgers Pakistan have prioritized building community support through a series of strategies as an essential component of their programs (Figure 1).

#### Understanding the Local Context

**FIGURE 2.**
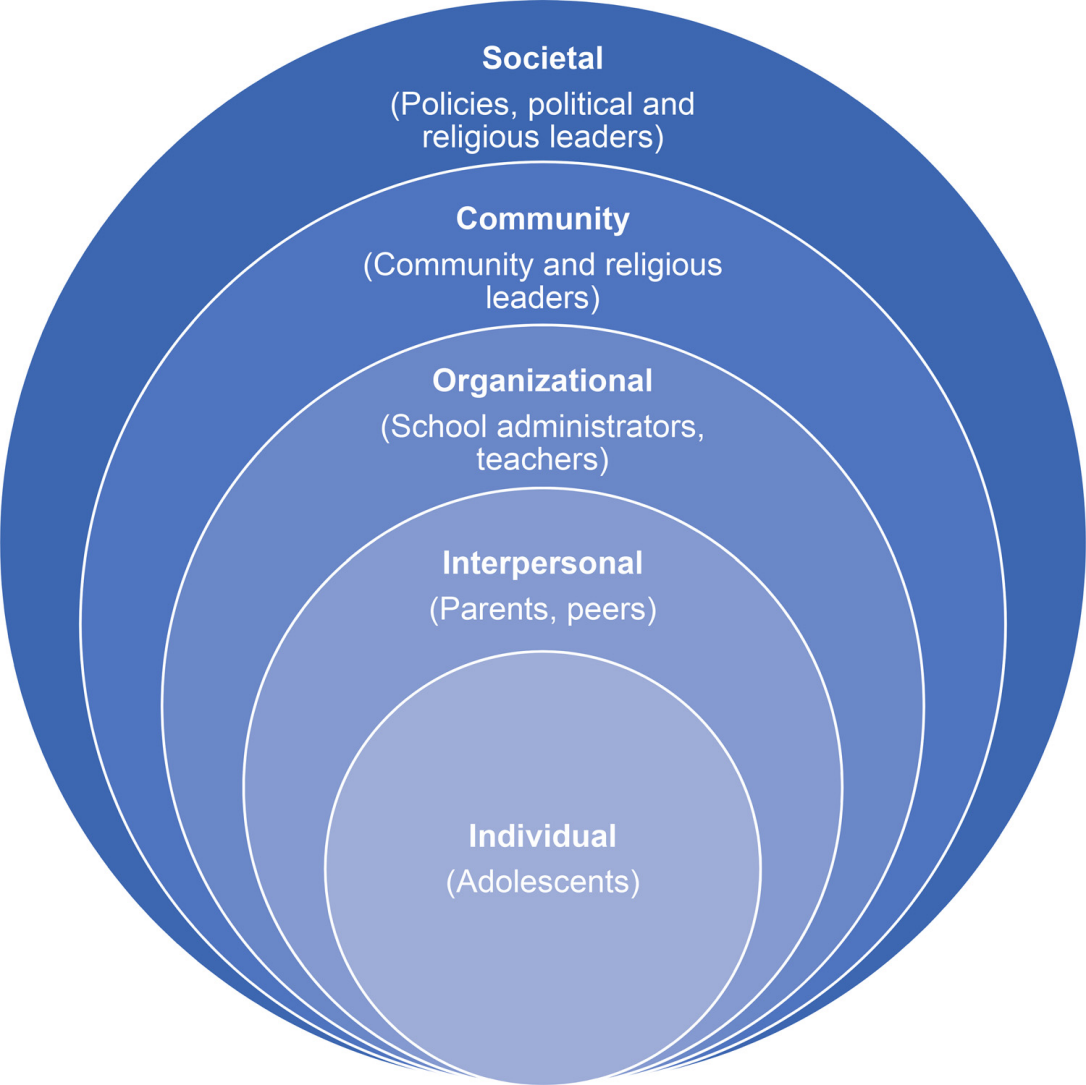
Ecological Framework: Levels of Adolescents' Influences

Before engaging with stakeholders to create the first adolescent-specific SRH curricula in Pakistan, Aahung carried out a power-mapping exercise to identify key decision makers in the lives of adolescents. This exercise revealed various levels of influence within an adolescent's environment, in line with an ecological framework, that needed to be understood in order to sensitize and engage influencers to successfully reach adolescents with sexuality education (Figure 2). The exercise also included a series of communication-focused activities, such as learning forums and in-person meetings, to secure buy-in from groups at the organizational, community, and provincial and national levels, ranging from local religious groups and school associations to the School Education and Literacy Department. At the same time, Aahung conducted a context evaluation, with specific attention to existing vulnerabilities and access to SRH information and services. This community-based research, which identified critical influencers, health and social problems, gaps in adolescents' SRH knowledge, and myths and misconceptions that contribute to inequitable gender norms, served as the foundation of the program's content development.

Aahung carried out community-based research, which served as the foundation of the program's content development.

#### Designing and Framing the Curricula With Careful Consideration of Context and Sensitive Topics

After consulting with their communities, Aahung recognized it would be culturally inappropriate to directly address sensitive topics like premarital sexual activity or adolescent contraceptive use. Additionally, they recognized the stigma associated with the term “sexuality education.” Instead, they labeled their program as life skills-based education (LSBE), which is an interactive teaching methodology that informs students about health while equipping them with skills to better manage their own lives and make healthier decisions.^4^ They adapted the World Health Organization's key guidelines for LSBE,^14^ UNESCO's *International Technical Guidance on Sexuality Education*,^15^ and additional curricula resources, such as the International Planned Parenthood Federation's *It's All One Curriculum*,^16^ to the local context.

Aahung targets related challenges identified as problems—including child marriage and gender-based violence—by local actors themselves, thus navigating the minefield of cultural sensitivities that frequently halt action. Their curricula use only widely acceptable terminology, were pilot-tested in schools and communities, and were reviewed by an advisory group of parents, educators, school administrators, religious scholars, and members of the Sindh School Education and Literacy Department (Box 2). In this way, Aahung simultaneously serves community interests and adheres to internationally established recommendations by focusing on common intermediate outcomes that contribute to positive SRH behaviors, such as comfort with one's own body, communication skills, confidence, and decision-making abilities, with specific attention to issues related to power and gender (e.g., questioning why fathers usually make important financial decisions for the family, or why boys are expected to do better in certain academic subjects than girls). If more explicit or sensitive questions arise during discussions, teachers are trained to provide accurate information. This approach is backed by supportive evidence: a recent report found that programs that addressed gender or power were 5 times more likely to be effective at decreasing rates of sexually transmitted infections or unintended pregnancy as those that did not.^17^

Programs that address gender or power are more effective at decreasing rates of sexually transmitted infections or unintended pregnancy.

BOX 2 **Deciding on the Curricula's Inclusion or Exclusion of Religious Content**

As a strategic choice, Aahung decided to maintain a purely secular, human-rights based program during the consultation process with religious scholars, despite pressure from the government to include religious content in the curriculum. Aahung believed that the acceptance of the program should be led by schools and parents, not religious groups, and were therefore cautious with their allocation of decision-making power. Additionally, they hoped this decision would ensure that the program could be defended purely based on human rights and prevent unnecessary politicization of the content. However, Aahung did collaborate with religious scholars to ameliorate contentious issues through tactful emphasis and phrasing. For example, while there was initial pushback on discussion of condom use in the chapter on HIV/AIDS, Aahung helped the scholars understand the significance of condoms, specifically as a critical tool for preventing HIV to promote health and well-being among sexually active individuals. A subsequent point of contention emerged when the scholars asserted that the recommendation for condom use should explicitly be made in reference to married couples. In response, Aahung restructured the chapter to simply discuss unsafe sexual intercourse, without specifying its relevance for married *or unmarried* individuals. Through the years, Aahung has continued to revise content based on emerging issues and revised laws and policies (e.g., Sindh changed the minimum age of marriage from 16 to 18).

Following backlash by certain religious groups and the media in parts of Pakistan in 2011, Rutgers Pakistan decided to engage religious scholars to provide support for key messages in life skills-based education through teachings from the Quran and Hadith. Relevant references to the Quran were included in the workbooks and teachers' manual, which helped to demonstrate that the Quran is, in fact, more progressive on topics related to relationships and growing up than was commonly perceived in communities.

Building on existing sexuality education content from programs elsewhere, Rutgers Pakistan's curricula materials also underwent several consultative processes to tailor the content to the target audience and the local context, as informed by relevant stakeholders. The program was adapted to the LSBE methodology, and it introduced content on abuse, protection, child marriage, and gender (and later, gender-based violence). The revised curriculum was then pilot-tested with adolescents in Pakistan to ensure that the content was understandable and relevant to their needs. Additionally, after the decentralization of political power to the provincial level in 2010, Rutgers Pakistan consulted education departments, religious scholars, civil service organizations, media personnel, and young people and their networks to determine whether province-specific content was necessary. This process led to the alignment of the content and key messages in all provinces, along with the inclusion of different illustrations and designs to complement specific elements of each province's social and cultural contexts.

Lastly, Aahung and Rutgers Pakistan tailored their curricula to appropriately respond to the evolving cognitive and social development of their students, based on school and community demands. For example, Aahung's curriculum proceeds from a focus on body protection and communication skills in early primary school years, to content on pubertal changes, harassment, and negotiation and conflict resolution skills in middle school years, to more specific reproductive health topics in secondary school years, with an overarching emphasis on understanding and navigating gender and power dynamics. Additionally, Aahung developed content to specifically address the needs of older out-of-school adolescents (over age 15).

#### Engaging Gatekeepers Through Outreach and Sensitization

Successfully reaching adolescents also requires engaging their influencers or “gatekeepers”—in their immediate lives and at higher levels of the ecological framework (Figure 2). To accomplish this goal with interpersonal-level influencers, Aahung supported the sensitization and counseling of gatekeepers, such as parents and community members, by school administrators and teachers to improve engagement and transparency of the program. This strategy also served to proactively address layers of potential resistance. To increase outreach, Aahung held public theater performances, *melas* (fairs), mobile cinema campaigns, and discussion sessions to demystify LSBE and demonstrate its benefits. They also invited parents to school discussions about the importance of adolescent health, where Aahung and partners explicitly asked their permission to teach LSBE topics.

Similarly, Rutgers Pakistan created a Parent Involvement Strategy in 2011 to develop parents' interest in their children's education and to enhance communication and transparency of the program's content and objectives. Their evaluations indicated that parents generally viewed the LSBE program positively and noted improvements in parent-child communication.^11^ Rutgers Pakistan also trained men as Fathers for Change, disseminated “responsible fatherhood” and gender-equality messages through radio and social media, hosted events to celebrate International Father's Day, and conducted theater plays aimed specifically at men and boys. The focus on men and boys has been well received, with many men thanking the program for explicitly including them in their considerations. Integrating adolescents' parents in these early stages allowed Aahung and Rutgers Pakistan to create a positive impression and a foundation for future engagement and transparency of their programs.

Rutgers Pakistan created a strategy to involve parents and also focused on engaging men and boys specifically.

Finally, to secure support from critical gatekeepers at the community and societal levels and to attain institutional support from key power structures, Aahung conducted an extensive national power-mapping exercise. The exercise revealed key advocates and decision makers among influential government officials, as well as relevant processes and policies. Through subsequent advocacy efforts, including lobbying stakeholders, meeting face-to-face, coordinating learning forums, circulating position papers, and tailoring messages on an ongoing basis, Aahung garnered commitments from the Sindh School Education and Literacy Department, Board of Curriculum, Sindh Education Foundation, and the Sindh Private School Association.^4^ To harness the collective benefits of international and local advocacy efforts, it has similarly cultivated partnerships with organizations that share aligned objectives, such as the United Nations Population Fund, International Women's Health Coalition, Family Planning Association of Pakistan, Rozan, World Population Foundation, Shirkat Gah, and Oxfam Novib.

Aahung conducted a national power-mapping exercise to identify key decision makers and gain their support through advocacy efforts.

Likewise, Rutgers Pakistan's strategy has focused on consistently engaging community members, religious leaders, and government stakeholders in the education and health departments in ongoing learning and advocacy forums to create an enabling environment for all young people to exercise their rights. They have partnered with local civil service organizations, such as the Health and Nutrition Development Society in Sindh, AwazCDS-Pakistan in Punjab, and Participatory Integrated Development Society in Balochistan.^11^ By prioritizing evidence generation through operational research, Rutgers Pakistan has been able to share the tangible value of their initiatives with stakeholders in communities and the government. Advocacy efforts such as these have contributed to the inclusion of references to LSBE in the National Education Policy of 2009 and the inclusion of information about HIV/AIDS in the national curriculum for grades 9 and 10.^11^

Advocacy efforts by Rutgers Pakistan contributed to the inclusion of life skills-based education in the National Education Policy of 2009.

#### Institutionalizing the Programs

Using the goodwill generated through the continued engagement of key stakeholders, both Aahung and Rutgers Pakistan implemented their LSBE programs within the school system and worked closely with school administrations and teachers. By embedding their efforts within already accepted institutions and drawing credibility from their partners, the organizations accelerated the general acceptance of their LSBE programs. For Aahung, this process involved ongoing diplomacy and partnership building with school administrators, teachers, and school communities. They began by leveraging key advocates among government officials and civil service organizations to sensitize school decision makers on the utility of LSBE to address young people's health and social needs. Once a partnership was formed with schools, teachers—key determinants of successful implementation—were carefully selected based on their aptitudes and willingness to teach LSBE topics.^4^ Aahung then provided an intensive 5-day training-of-trainers course on comprehensive LSBE topics and participatory teaching methodologies, complete with teachers' guides, workbooks for students, and designated content delivery and skill-building practice sessions. These activities were complemented with on-site teaching support, advanced teacher trainings on skills such as counseling, and annual refresher trainings. Aahung's model of sustainability—grounded in its integration with existing systems and its front-loaded financial model predominantly based on human resource investments—has allowed more than 70% of the program's partners to operate independently.^4^ As another critical component of the program's ability to scale up and be sustainable, Aahung is working with the School Education and Literacy Department, the Textbook Bureau, and the Bureau of Curriculum to convert the current LSBE curriculum, which was designed to be taught as a stand-alone subject, to a framework that can be integrated with approved subject areas already taught in all public schools.

Aahung's model of sustainability is grounded in its integration with existing systems.

### How Aahung and Rutgers Pakistan Overcame Resistance

Despite Aahung and Rutgers Pakistan's best efforts to preempt resistance to their programs, the media and religious leaders both emerged as forces of resistance. In 2011 and 2012, conservative media outlets linked to a religious political party, Jamaat-e-Islami, criticized Rutgers Pakistan for “breaking the moral fabric of Pakistan” and corrupting the minds of students by promoting vulgarity. Following parliamentary discussions, its work was stopped in Punjab and in Sindh, where the government required that religious scholars vet the content. Aahung also experienced backlash ranging from religious disapproval and media pushback to misunderstandings from schools and communities about intentions and content.

When the media leveraged attacks, both NGOs strengthened their media presence to build positive public perception of their work and discredit false statements. Rutgers Pakistan reached out to a small group of respected and well-known journalists from print, radio, and television to facilitate dialogue with mass media personnel in the affected provinces. This stimulated public discussion of the utility of LSBE in addressing the vulnerabilities of adolescents and helping the country reach national education and health goals. Currently, Aahung is developing a more detailed risk management strategy to emphasize consistent engagement of media by building a network of supportive media personnel.

Rutgers Pakistan stimulated public discussion by reaching out to a small group of respected and well-known journalists.

As part of their media engagement strategies, both organizations relied on schools to demonstrate the positive impact of their programs on the confidence and performance of students and teachers. Because teachers and school administrators had been engaged with the LSBE programs from the beginning of their implementation, they served as a strong support base, with substantial buy-in for the programs' success. Rutgers Pakistan organized school visits for journalists, who then produced a number of positive stories about their firsthand observations. Aahung led sensitization and value clarification workshops with schools and the media and created an additional active mechanism, which included organizing specific meetings to discuss issues, to provide support to teachers and school administrators when cases of opposition arose by parents or the community. Furthermore, Aahung reviewed language, increased transparency, leveraged strategic partnerships, and engaged “champions” when faced with backlash.

Rutgers Pakistan and Aahung also took advantage of key moments of positive momentum when society would be more receptive to their messages. For example, Aahung strategically used opportune moments, such as during Ramadan or after widely publicized reports of gender-based violence emerged, to stress the value of their work and thereby increase community acceptance. Rutgers Pakistan used a resurgence of positive media attention to arrange for progressive religious scholars to review the content of their LSBE curriculum and supplement its content with messages from the Quran. This work fed into a series of meetings with parliamentarians, policy makers, religious scholars, and media personnel in Balochistan, whereby Rutgers Pakistan was able to present positive results of the LSBE programs to the provincial Secretary of Education and the Bureau of Curriculum. Rutgers Pakistan then shared all of the program materials with the Bureau of Curriculum, the Provincial Institute of Teacher Education, and the School Education and Literacy Department, who formed a committee to review the materials and encourage institutional ownership of the programs. Rutgers Pakistan supported this review by providing explanations and acceptable recommendations, and later assisted public-sector textbook writers in drafting a module for LSBE.^11^ The module was reviewed by the committee and approved by the Secretary of Education.^11^ Through perseverance, willingness to collaborate, and patience during changing administrations, these efforts culminated in a letter of recommendation by the Bureau of Curriculum to include LSBE in provincial textbooks.^11^

Aahung increased community acceptance by strategically using opportune moments to stress the value of their work.

Rutgers Pakistan assisted textbook writers in drafting a module for life skills-based education, resulting in its inclusion in provincial textbooks.

## DISCUSSION

In our review of the development of Aahung and Rutgers Pakistan's LSBE programs, we found 2 themes that improved uptake and positive reception of sexuality education: (1) building community support, and (2) responding to resistance. Within these themes, strategies included collaborating with leaders to select issues to address, tactfully framing issues with careful consideration for sensitivities, engaging adolescents' influencers, strengthening media presence, showcasing school programs to increase transparency, and choosing critical times to introduce messages. The success of Aahung and Rutgers Pakistan was grounded in their readiness to understand the nuanced context within the communities, to collaborate with many groups of stakeholders at different levels of the ecological framework—including parents, school officials, religious leaders, media personnel, and adolescents themselves—to ensure support, and to stand up to forces of resistance to pursue their collective goals. Additionally, both programs have noted areas of weakness, such as the lack of detailed content on sexual diversity and sexual behavior (especially related to contraception and abortion), the need for expanded work with parents and other community members to create a truly enabling environment for adolescents to exercise their rights, and the challenge of securing sufficient buy-in from government and schools to ensure meaningful institutionalization, which will guide future planning and action.

Building community support and responding to resistance improved uptake and positive reception of sexuality education.

The value of comprehensive sexuality education for adolescents is well established, and past studies have collected data on the effective characteristics of sexuality education programs around the world.^18^ The counter-arguments to common points of resistance to sexuality education have been clearly outlined by numerous sources.^18^ Additionally, resistance has been identified by many sources as a key obstacle in scaling up sexuality education in diverse contexts.^6^^–^^8^^,^^19^ Too often, however, the conversation stops there. There has been very little discussion on specific programmatic strategies to proactively prepare for backlash and respond timely and effectively.

We recognize that this review is limited to Pakistan and the work of only 2 NGOs, but it is an important contribution because resistance to sexuality education has limited coverage in the literature. We obtained data from key informants at Aahung and Rutgers Pakistan, who provided valuable insights to our analysis. We acknowledge that our analysis could also benefit from the perspectives of other community groups.

The implication of our findings is that even in conservative contexts, we can find ways to build support for sexuality education and stand up to forces of resistance. It takes an extraordinary amount of local knowledge, work, and courage to do so, which potentially explains why we have not seen more progress on SRH education. In the specific context of Pakistan, Aahung and Rutgers Pakistan remained cognizant of the fact that resistance comes in many shapes and forms. There can be general resistance due to the discomfort of discussing sensitive topics such as adolescent SRH. There can be logistical resistance due to resource constraints and a lack of adequately trained professionals to carry out and manage the programs. Lastly, there can be conservative resistance to the basic premise of LSBE, the idea that young people should receive any information pertaining to reproductive health, and the perception of “western” ideological influence. As described, the success of the 2 NGOs is grounded in the understanding that preventing and responding to each form of resistance requires specific strategies and contextual knowledge. There is no one-size-fits-all approach to building community support and responding to resistance. In this case, Aahung chose to use a rights-based approach to bring legitimacy to its work, while Rutgers Pakistan supplemented its content with messages from the Quran, as informed by a review with progressive religious scholars. We need further research on diverse strategies to build community support and respond to resistance in a variety of settings, and some combination of these strategies must be an integral part of successful sexuality and reproductive health education programs.

Even in conservative contexts, we can build support for sexuality education and stand up to resistance.

## CONCLUSION

The experiences of Aahung and Rutgers Pakistan in the promotion of SRH education are inspiring and their approaches to identifying strategies that worked in their context can inform programs in countries around the world. They recognized that it is not enough to run effective education programs if the programs are not accepted locally and by society at-large. Both organizations know they must be ready to respond to occasional backlash (often coordinated) from media, religious institutions, and other groups. A 2-pronged approach, whereby these organizations reached out to local communities while simultaneously working with the media and religious institutions, secured local support for both organizations as well as a network of journalists and community leaders ready to champion their cause in the face of heated opposition. We call for further sharing of challenges, specifically related to resistance, with sexuality education programs in order to develop a toolbox of additional strategies for community uptake.

## Notes

**Peer Reviewed**

**First Published Online:** February 14, 2018

**Funding:** This work was funded by the UNDP-UNFPA-UNICEF-WHO-World Bank Special Programme of Research, Development and Research Training in Human Reproduction (HRP), a cosponsored program executed by the World Health Organization (WHO).
